# Iodine-125 Brachytherapy Prophylaxis after Radiofrequency Ablation Cannot Benefit Patients in High Risk of Locoregional Hepatocellular Carcinoma Recurrence

**DOI:** 10.1038/s41598-017-03831-5

**Published:** 2017-06-16

**Authors:** Jian-Fei Tu, Ya-Hui Ding, Li Chen, Xi-Hui Ying, Deng-Ke Zhang, Fa-Zong Wu, Zhong-Wei Zhao, Jian-Song Ji, Wang-Gang Zhang, Hai Zou

**Affiliations:** 1 Department of Radiology, The Fifth Affiliated Hospital of Wenzhou Medical University, Wenzhou, 325000 Zhejiang Province China; 2 Department of Cardiology, Zhejiang Provincial People’s Hospital, Hangzhou, 310000 Zhejiang Province China; 3 Institution of Drug Clinical Trials, Zhejiang Provincial People’s Hospital, Hangzhou, 310000 Zhejiang Province China

## Abstract

This study evaluated if iodine-125 brachytherapy prophylaxis after radiofrequency ablation (RFA) prolongs time to recurrence (TTR) and overall survival (OS) of patients in high risk of locoregional hepatocellular carcinoma (HCC) recurrence. 116 patients with total tumor necrosis after RFA were divided into iodine-125 brachytherapy prophylaxis treatment group and control group. The primary endpoint was TTR, and secondary endpoints were OS and treatment-related adverse events. There were no significant differences among the baseline characteristics of two subgroups patients. The mean iodine-125 particles were 29.8 (26.59 ± 12.51 mCi) per patient. The mean follow-up was 25 months, and mean TTR of treatment and control groups were 21.7 and 15.9 months (*P* = 0.733); mean OS of two subgroups were 41.7 and 40.9 months (*P* = 0.316). There were no significant differences of 1-, 2-, 3-, 4-and 5-years TTR and OS and patients’ immunity pre- and 1 month post-treatment. Extrahepatic metastasis was found to have a statistically significant influence on TTR, and AFP, extrahepatic metastasis were found to have a statistically significant influence on OS by multivariate analysis. There was no major complications and procedure related death. Iodine-125 brachytherapy prophylaxis after RFA can’t improve TTR and OS of HCC patients who were in high risk of locoregional tumor recurrence.

## Introduction

Radiofrequency ablation (RFA) has been shown to be a safe and efficient treatment for hepatocellular carcinoma (HCC)^1–3^. However, clinical studies have reported a high rate of inadequate RFA treatment for large tumors (>3 cm) and high locoregional tumors recurrence rate^4, 5^. The long term prognosis, however, remains unsatisfactory because of frequent development of locoregional tumor recurrence^6^. Especially to the patients who with irregular liver lesions, or the tumor locate adjacent to liver capsule, diaphragm, gallbladder, and important blood vessels^7^. Hirooka, *et al*.^7^ reported that the median time to recurrence of patients with HCC in the tumor blood drainage area following RFA was significantly shorter than patients who with HCC not in the tumor blood drainage area (434 days *vs* 1,474 days; *P* = 0.0037), and the cumulative locoregional tumors recurrence rates at 1, 3, and 5 years post-operatively were 0, 0, and 1.5%, *vs* 3.8%, 17.0%, and 22.8% (*P* < 0.0001), respectively. Tumor lesions locate adjacent to liver capsule, diaphragm, gallbladder, and important blood vessels has a high rate of inadequate RFA, and those patients were in high risk of tumor recurrence after RFA^8, 9^.

Iodine-125 brachytherapy can get high locoregional tumors control and low complication rate in the treatment of malignant tumors^10–14^. It was reported that adjuvant iodine-125 brachytherapy can significantly prolong time to recurrence (TTR) and increase the overall survival (OS) of patients who with HCC^15^.

The purpose of this study was to evaluate if iodine-125 brachytherapy prophylaxis after RFA prolongs TTR and OS of HCC patients who were in high risk of locoregional tumor recurrence.

## Materials and Methods

### Study Design

This retrospective study was approved by the institutional review board and was performed in accordance with the ethical standards as laid down in the 1964 Declaration of Helsinki. All patients who received iodine-125 brachytherapy prophylaxis after RFA at our institution were included. Cases were identified through departmental procedural logs. Patient demographics, clinical information, and procedural data were gathered from patients’ medical records.

### Patients

Inclusion criteria were: age between 18–80 years; clinic or histologically proven HCC; no portal vein tumor thrombus; the tumor lesions were locate adjacent to liver capsule, diaphragm, gallbladder, or important blood vessels; patients who with total tumor necrosis after RFA; Karnofsky performance status (KPS) > 70; Child-Pugh A or B; adequate hepatic function (albumin > 2.5 g/dL, total bilirubin < 3.0 mg/dL), and complete clinic follow-up.

Exclusion criteria were: severe cardiac, pulmonary, cerebral, or renal dysfunction; with other active malignancy; any other contraindication like: gastrointestinal hemorrhage in the past month, refractory ascites, encephalopathy, impaired coagulation, severe portal hypertension; contraindications to lidocaine; patients who received other treatment pre RFA; patients with a history of concomitant use of some targeting agents, chemotherapy and immunotherapy; and patients who were lost to follow-up. Informed consent was obtained in all patients, and the study was approved by the Ethics Committee of Lishui Central Hospital (Lishui, China). All the methods were carried out in accordance with the approved guidelines.

### Groups and Definitions

Patients were divided into two subgroups, including treatment group: patients underwent iodine-125 brachytherapy prophylaxis after RFA; and control group: only follow-up. The primary endpoint was TTR, and the secondary endpoints were OS and treatment-related adverse events.

High risk of locoregional HCC recurrence was defined as tumor lesions locate adjacent to liver capsule, diaphragm, gallbladder, and important blood vessels. TTR was measured from the date of first RFA to the date when the diagnosis of locoregional tumors recurrence was established (only locoregional tumor recurrences were calculated, and the distant recurrences were excluded). Patients who died without recurrence were censored at their date of death. OS was calculated from the date of first RFA to the time of death or the last follow-up.

### Techniques

RFA technique was described in our previous study^16^. Enhanced computed tomography (CT) or magnetic resonance imaging (MRI) was underwent 4–6 weeks after the procedure. Patients who with the tumor locate adjacent to liver capsule, diaphragm, gallbladder, important blood vessels, and identified total tumor necrosis after RFA, were divided into treatment and control groups. Patients of treatment group received iodine-125 brachytherapy prophylaxis.

Iodine-125 particles (0.8 mm in diameter and 4.5 mm in length, Tianjin Saide Bio-Pharmaceutical Co. Ltd. Tianjin, China) were implanted under CT guidance. The radioactivity of each iodine-125 seed is 25.9 MBq with a half-life of 59.4 days. The principal photon emissions are 27.4–31.4 keV X-ray and 35.5 keV γ-ray. The half-value thickness of tissue for iodine-125 seeds is 17 mm, and the initial dose rate is 7 cGy/h. The effective irradiating range is 20 mm. The radioactivity per seed ranged from 0.8 to 1.0 millicuries (mCi).

Iodine-125 particles were implanted in the place of high risk of tumor recurrence according to the tumor location. The number of the iodine-125 particles was determined by the area of tumor margin near the liver capsule, diaphragm, gallbladder, or important blood vessels. The distance between iodine-125 particles was within 0.5–1.0 cm according to the literature^17, 18^.

Repeated RFA was applied to the patients who was diagnosed of locoregional tumor recurrence. Iodine-125 brachytherapy with or without transarterial chemoembolisation (TACE) was carried out if the locoregional tumor was unsuitble for repeat RFA.

### Evaluation and Clinical Follow-Up

The evaluation criteria of imaging mass as “viable tumor” was the evaluated object as proposed by American Association for the Study of Liver Diseases (AASLD), which is modified Response Evaluation Criteria In Solid Tumors criteria (mRECIST)^8^. After discharge, outpatient clinic visits were offered every month at first 3 months, and every 3 months thereafter. More frequent evaluations were necessary when indicated. Symptom status, complete blood count, liver function, alpha-fetoprotein (AFP), and enhanced CT scan or MRI were obtained during follow-up.

### Statistical Analysis

The statistical analyses were performed by using SPSS 23.0 software (SPSS, Chicago, Ill). Comparisons between the two subgroups were performed by using the Student *t* test for continuous data and the Chi-square test for categorical data. TTR and OS were calculated by using the life-table method. Survival curves were constructed by using the Kaplan-Meier method and were compared by using the log-rank test. The relative prognostic significance of the variables in predicting the OS rate and the time to tumor recurrence or metastasis were assessed by using multivariate Cox proportional hazards regression analysis and logistic regression analysis, respectively. All statistical tests were two sided, and *P* < 0.05 was considered to indicate a significant difference.

## Results

### Patients and treatment-related data

**Figure 1:**
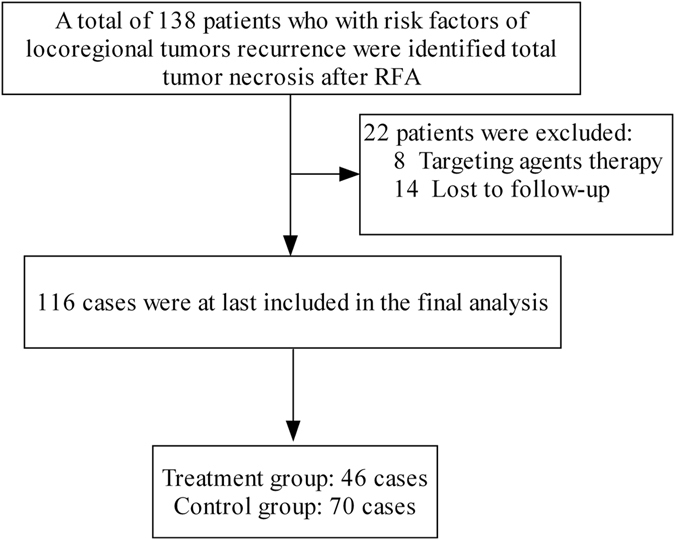
Selection of the patients.

From Feb 2009 to Aug 2016, a total of 138 HCC patients who with the lesions locate adjacent to liver capsule, diaphragm, gallbladder, or important blood vessels, were identified total tumor necrosis after RFA. Of the 138 cases, a total of 22 cases were excluded for received targeting agents therapy (n = 8), lost to follow-up (n = 14), and a total of 116 cases were at last included in the final analysis, including 46 patients (42 men, 4 women; mean age, 59.1 ± 10.4 years; range, 29–78 years) of treatment group, and 70 patients (62 men, 8 women; mean age, 56.2 ± 11.8 years; range, 18–80 years) of control group (Fig. 1). Table 1 lists the characteristics of the 2 subgroups patients. There were no significant differences among the baseline characteristics of the two subgroups patients.

**Table 1:** Baseline characteristics of the 2 subgroups patients.

Characteristics	RFA+^125^I (n = 46)	RFA (n = 70)	*p* value
Age (years)	59.1 ± 10.4	56.2 ± 11.8	0.175
Sex			0.872
Male	42	62	
Female	4	8	
HBV			0.592
Yes	41	60	
No	5	10	
AFP (ng/mL)			0.963
≤400	35	53	
>400	11	17	
No. of lesions			0.205
1	22	41	
2	3	8	
3	21	21	
Diameter of tumor (cm)			0.294
between 3–5	32	42	
≤3	14	28	
Diameter of main lesions (cm)	3.3 ± 2.4	3.4 ± 2.6	0.710
Tumor location			0.614
Adjacent to liver capsule	21	38	
Adjacent to gallbladder	16	22	
Adjacent to vital vessels	9	10	
Extrahepatic metastasis			0.926
Yes	5	8	
No	41	62	
Child-Pugh classification			>0.999
A	42	64	
B	4	6	
KPS	90.2 ± 3.9	89.6 ± 6.0	0.954

HBV: hepatitis B virus; AFP: alpha-fetoprotein; KPS: karnofsky performance status.

Treatment group: a total of 1223 iodine-125 particles (29.8 iodine-125 particles and 26.59 ± 12.51 mCi per case) were used through 20 iodine-125 procedures (one iodine-125 procedure per case). Of the 46 patients who received iodine-125 brachytherapy prophylaxis, abdominal pain and fever was observed in 16 patients and none required medical intervention. There was no major complications and procedure relate death.

### Outcomes

**Figure 2:**
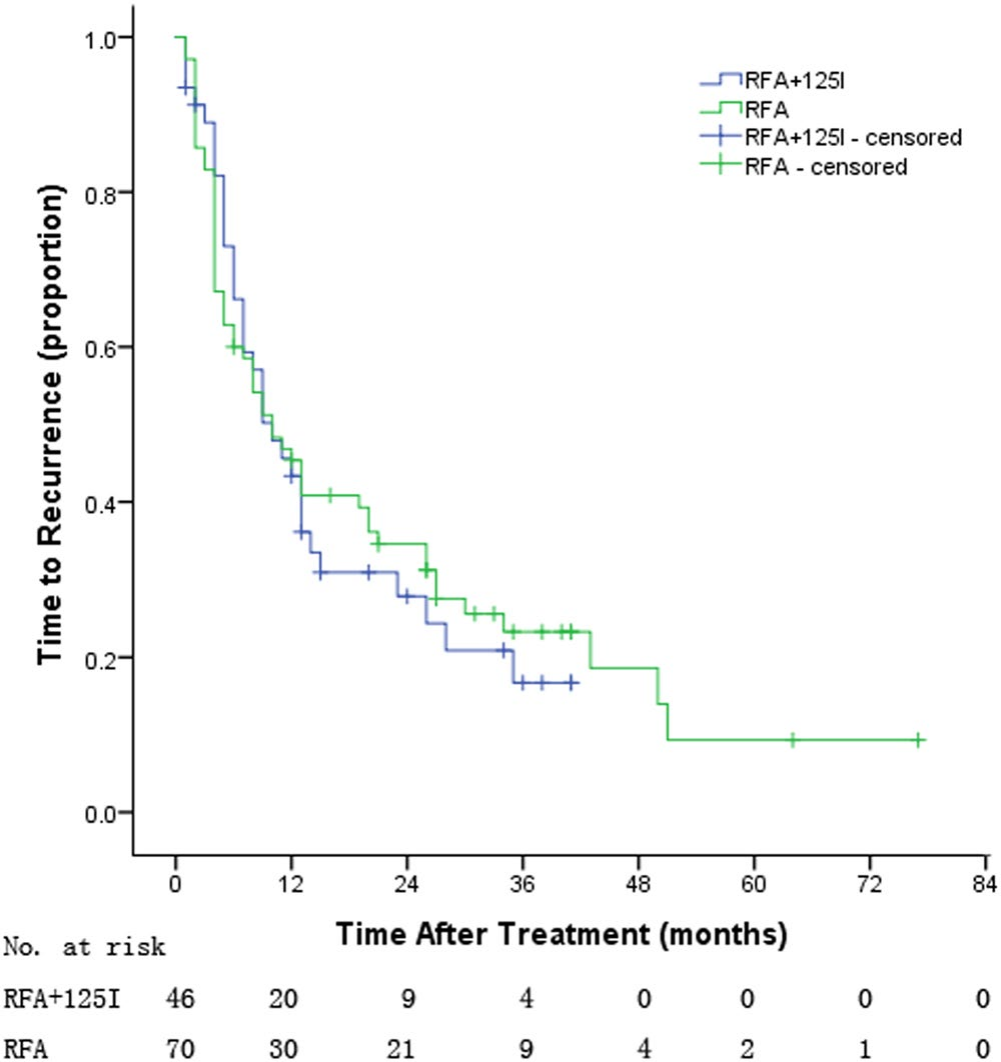
The time to recurrence of the two groups patients.

**Figure 3:**
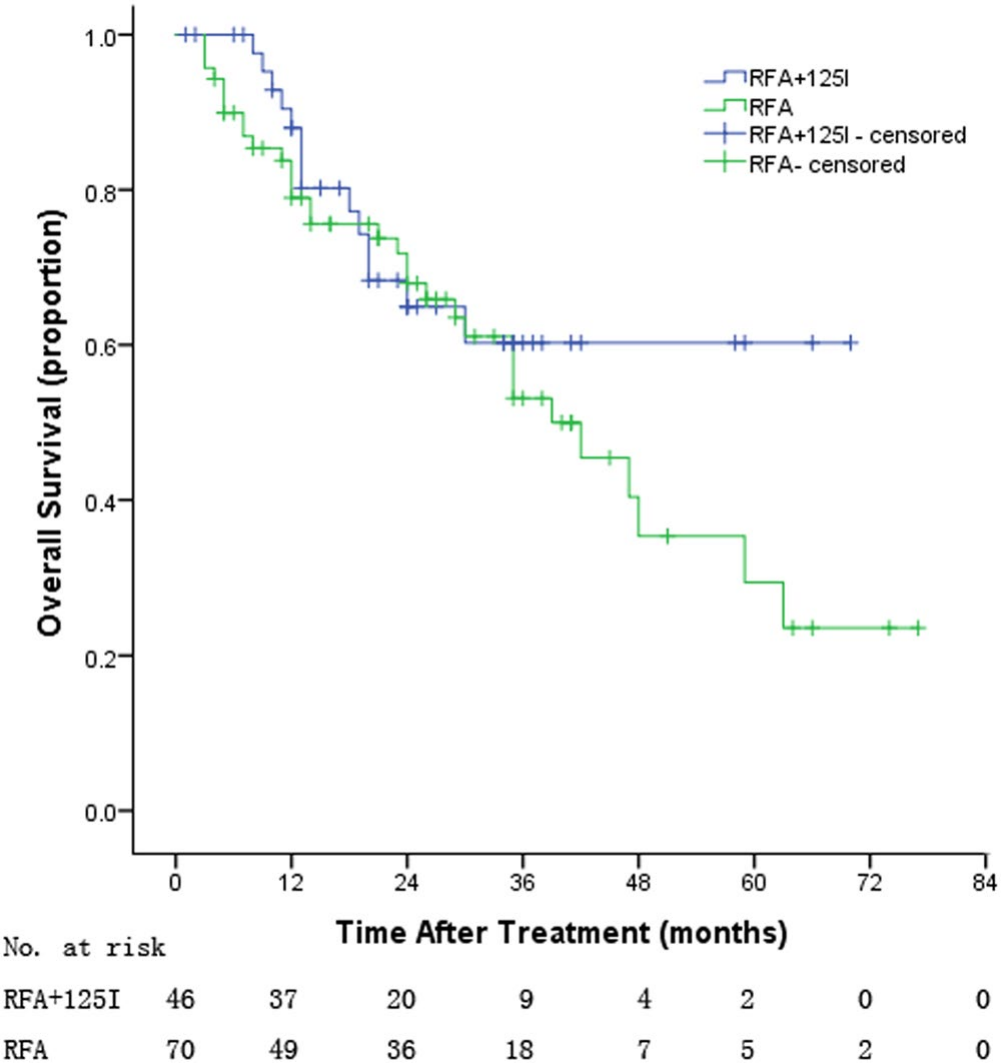
The overall survival of the two groups patients.

The mean follow-up was 25 months (range, 1–77 months), and the mean TTR of treatment and control groups were 21.7 and 15.9 months, respectively (*P* = 0.733, Fig. 2); and the mean OS of treatment and control groups were 41.7 and 40.9 months, respectively (*P* = 0.314, Fig. 3). Table 2 lists the 1-, 2-, 3-,4- and 5-years TTR and OS, and there were no significant differences between the two groups (*p* > 0.05).

**Table 2:** The TTR and OS of the 2 subgroups patients.

TTR and OS of the 2 subgroups patients										
Outcomes	Survival Analysis									
	TTR					OS				
	RFA+^125^I	RFA	*P* value	HR	95%CI	RFA+^125^I	RFA	*P* value	HR	95%CI
No. of patients	46	70	0.7326	1.082	0.6875–1.704	46	70	0.3164	0.7342	0.4012–1.344
1-year survival	43.3%	45.4%				80.2%	78.9%			
95%CI	28.7–58.0	33.6–55.7				67.9–92.5	69.1–88.8			
2-years survival	27.9%	34.5%				60.3%	67.9%			
95%CI	14.1–41.7	23.2–45.9				43.3–77.3	56.1–79.7			
3-years survival	16.7%	23.3%				60.3%	53.1%			
95%CI	3.8–29.6	12.5–34.0				43.3–77.3	39.1–67.1			
4-years survival	16.7%	18.6%				60.3%	35.3%			
95%CI	3.8–29.6	6.8–30.5				43.3–77.3	18.0–52.7			
5-years survival	16.7%	9.3%				60.3%	29.4%			
95%CI	3.8–29.6	1.6–20.2				43.3–77.3	11.6–47.3			

### Predictors of TTR and OS

Of the 7 clinical factors analyzed by Cox regression model, univariate analysis identified extrahepatic metastasis associated with TTR and 3 significant factors associated with survival, including AFP (*P* < 0.001), diameter of tumor (*P* = 0.044), and extrahepatic metastasis (*P* = 0.024). However, only extrahepatic metastasis (*P* = 0.036) was found to have a statistically significant influence on TTR, and AFP (*P* < 0.001), extrahepatic metastasis (*P* = 0.009) were found to have a statistically significant influence on OS by multivariate analysis (Table 3).

**Table 3:** Patient characteristics as determinants of TTR and OS.

Factors	TTR		OS	
	Univariate analysis *P* value	Multivariate analysis *P* value	Univariate analysis *P* value	Multivariate analysis *P* value
Sex	0.680		0.588	
HBV	0.069		0.976	
AFP(ng/mL)	0.129		<0.001	<0.001
No. of lesions	0.895		0.837	
Diameter of tumor (cm)	0.224		0.044	
Extrahepatic metastasis	0.027	0.036	0.024	0.009
Child-Pugh classification	0.486		0.761	

### Safety

There were no significant difference of the patients’ immunity, including IgG, IgA, IgM, IgE, C3, and C4 pre- and 1 month post-iodine-125 brachytherapy (Table 4).

**Table 4:** The immunity items pre- and 1 months post- iodine-125 brachytherapy of the 2 subgroups patiens.

	IgG	IgA	IgM	IgE	C3	C4
Pre-	15.02 ± 3.28	2.61 ± 0.71	1.38 ± 0.57	82.62 ± 110.19	1.15 ± 0.34	0.22 ± 0.10
Post-	14.99 ± 3.03	2.72 ± 0.64	1.41 ± 0.46	66.64 ± 66.78	1.16 ± 0.35	0.21 ± 0.07
*t*	0.067	−2.078	−0.356	0.674	−0.347	0.409
*P* value	0.948	0.060	0.728	0.513	0.735	0.690

## Discussion

The present study demonstrates iodine-125 brachytherapy prophylaxis after RFA cannot improve TTR and OS of HCC patients who were in high risk of locoregional tumor recurrence, with the mean TTR were 21.7 and15.9 months, and mean OS were 41.7 and 40.9 months of two subgroups, respectively.

RFA has emerged as a new curative treatment owing to its safety and effectiveness for early-stage small HCC^19^. However, the patients who with the tumor location close to blood vessels, liver capsule, and vital structures were in high risk of locoregional tumor recurrence^8, 9, 20, 21^. It was reported that the combination of RFA and TACE or percutaneous ethanol injection (PEI) in the management of HCC in high-risk locations has a slightly higher primary effectiveness rate than does RFA alone^9, 22^. Many other techniques can also improve the results of RFA for HCC in high-risk locations^23, 24^.

Iodine-125 brachytherapy can get high locoregional tumor control rate of malignant tumors^10–14^. Also, the adjuvant iodine-125 brachytherapy can significantly prolonged TTR and OS of patients who with HCC^15^. However, there was no report about the iodine-125 brachytherapy prophylaxis for patients who have risk factors of locoregional tumors recurrence. Can the iodine-125 brachytherapy prophylaxis after RFA prolongs TTR and OS of patients with HCC. This study was carried out to answer those questions.

The patients of the current study were all in high risk of locoregional tumor recurrence. The results of the current study proved that although the TTR and OS of treatment groups patients were longer than the control groups patients, there were no significant differences among them. The negative results of this study maybe have close relationship with the total tumor necrosis was achieved in all the treatment group patients one month after RFA, the disease was stable of such patients and the iodine-125 brachytherapy prophylaxis cannot benefit such patients. The results of this study proved that iodine-125 brachytherapy prophylaxis after RFA cannot benefit the patients who were in high risk of locoregional tumor recurrence.

A number of factors that have been reported to limit the TTR and OS for patients with HCC. Our study further reveals that extrahepatic metastasis (*P* = 0.036) to be independent prognostic factors for TTR, and AFP (*P* < 0.001), extrahepatic metastasis (*P* = 0.009) to be independent prognostic factors for OS.

Iodine-125 brachytherapy has been proved to a safe manner in the treatment of malignant tumors. Recently, Iodine-125 brachytherapy has also been used for treating HCC, and it has a larger local irradiation dose and results in less damage to normal liver tissues^8, 9^. A possible reason for this is that the irradiation has a stronger therapeutic effect on the thermal-damaged lesions due to the synergistic effect of radiotherapy and thermotherapy. More importantly, the killing radius of ^125^I is short (1.7 cm), which almost has no effect on the normal tissues around the lesions^18, 25^. Patients of this study were in high risk of locoregional tumor recurrence, and received iodine-125 brachytherapy. The results of this study proved iodine-125 brachytherapy is safe after RFA.

### Study limitation

The major limitation of this nonrandomized study is that its retrospective nature. Also, the amount of patients and the observation time was short. Furthermore, there were many factors, such as treatment of the locoregional tumors recurrence, metastasis, may influence on the overall prognosis, which might bias the results.

## Conclusion

Iodine-125 brachytherapy prophylaxis after RFA cannot improve TTR and OS of HCC patients who were in high risk of locoregional tumor recurrence.
